# miR-26a promotes axon regeneration in the mammalian central nervous system by suppressing PTEN expression

**DOI:** 10.1093/abbs/gmab044

**Published:** 2021-04-23

**Authors:** Jing Zhang, Yan-Xia Ma, Yu-Qiang Zeng, Shuang-Feng Zhang, Zhao-Qian Teng, Jun Gao, Saijilafu, Chang-Mei Liu

**Affiliations:** State Key Laboratory of Stem Cell and Reproductive Biology, Institute of Zoology, Chinese Academy of Sciences, Beijing 100101, China; Savaid Medical School, University of Chinese Academy of Sciences, Beijing 100049, China; Institute for Stem Cell and Regeneration, Chinese Academy of Sciences, Beijing 100101, China; Department of Orthopaedics, The First Affiliated Hospital of Soochow University, Orthopaedic Institute, Soochow University, Suzhou 215007, China; State Key Laboratory of Stem Cell and Reproductive Biology, Institute of Zoology, Chinese Academy of Sciences, Beijing 100101, China; Savaid Medical School, University of Chinese Academy of Sciences, Beijing 100049, China; Institute for Stem Cell and Regeneration, Chinese Academy of Sciences, Beijing 100101, China; State Key Laboratory of Stem Cell and Reproductive Biology, Institute of Zoology, Chinese Academy of Sciences, Beijing 100101, China; Savaid Medical School, University of Chinese Academy of Sciences, Beijing 100049, China; Institute for Stem Cell and Regeneration, Chinese Academy of Sciences, Beijing 100101, China; State Key Laboratory of Stem Cell and Reproductive Biology, Institute of Zoology, Chinese Academy of Sciences, Beijing 100101, China; Savaid Medical School, University of Chinese Academy of Sciences, Beijing 100049, China; Institute for Stem Cell and Regeneration, Chinese Academy of Sciences, Beijing 100101, China; Department of Neurosurgery, Peking Union Medical College Hospital, Chinese Academy of Medicine Sciences and Peking Union Medical College, Beijing 100730, China; Department of Orthopaedics, The First Affiliated Hospital of Soochow University, Orthopaedic Institute, Soochow University, Suzhou 215007, China; State Key Laboratory of Stem Cell and Reproductive Biology, Institute of Zoology, Chinese Academy of Sciences, Beijing 100101, China; Savaid Medical School, University of Chinese Academy of Sciences, Beijing 100049, China; Institute for Stem Cell and Regeneration, Chinese Academy of Sciences, Beijing 100101, China

**Keywords:** miR-26a, PTEN, optic nerve injury, optic nerve regeneration, retinal ganglion cell survival

## Abstract

The permanent disability after the central nervous system (CNS) injury is due to the weakened regeneration ability of the damaged axon, resulting in the loss of the rebuilding functional relationship with the original targets. One determinant of successful axon regeneration is the activation of intrinsic axon growth ability of injured neurons. MicroRNAs are important epigenetic factors controlling axon regeneration. Here, we demonstrated that the expression of miR-26a in hippocampal neurons is upregulated developmentally. Inhibitions of endogenous miR-26a suppressed the axon growth in hippocampal neurons, and overexpression of miR-26a promoted its axon growth. We also found that the overexpression of miR-26a in retinal ganglion cells also promoted retinal ganglion cells’ survival and optic nerve regeneration. Moreover, endogenous miR-26a promotes the hippocampal neuronal axon growth by suppressing phosphatase and tensin homolog deleted on chromosome ten (PTEN) expression. Thus, our results suggested that the miR-26a‒PTEN pathway regulates CNS axon growth. Collectively, the study not only reveals a new mechanism underlying mammalian axon regeneration but also expands the pool of potential targets that can be manipulated to enhance CNS axon regeneration.

## Introduction

Adult mammalian central nervous system (CNS) neurons have limited capacity to regenerate their axons after damage due to their weak capacity of intrinsic axon growth and the inhibitory microenvironment, which seriously impedes functional recovery [1]. Therefore, axonal regrowth is essential for the functional recovery after CNS injury [2]. Earlier studies mainly emphasized the role of extrinsic axon growth inhibitors [3]. Recent studies gradually highlighted the importance of neuron-intrinsic axon growth abilities [1]. Thus far, numerous axon growth–promoting or axon growth–inhibiting molecules in the CNS have been identified, such as the transcription factor SRY-box containing gene 11 (*Sox 11*) [4], Krüppel-like factors (*KLFs*) [5], and signal transducer and activator of transcription 3/suppressor of cytokine signaling 3 (*STAT3/SOCS3*) [6,7]. For example, Sox11 overexpression alone in mature neurons has been shown to sufficiently promote axon regeneration in the CNS [4], and Sox11 is the mechanistic target of the mTOR/PTEN pathway [8,9]. The overexpression of Klf6/7 can also effectively promote the regeneration of the optic nerve and corticospinal tract, and deleting Klf4 also enhances the optic nerve regeneration [5,10]. However, the long-distance axon regeneration *in vivo* is still limited, and clinical use may call for more alternative methods to enhance the intrinsic axon growth potential of injured CNS neurons.

In the CNS, non-coding ribonucleic acids (RNAs) such as microRNAs play an important role during neurogenesis [11,12] and in the synaptic function of mature neurons [13]. However, the roles of microRNAs in the regulation of neuronal morphogenesis are much less studied, especially in the regulation of mammalian axon regeneration after CNS injury.

Our previous study found an miR-26a‒GSK3β‒Smad1 signaling pathway regulating mammalian axon regeneration in the peripheral nerve system (PNS) [14]. Here, we tested whether miR-26a can regulate CNS axon regeneration or not. Our results showed that the expression level of miR-26a is gradually increased in hippocampus during development. Additionally, the overexpression of miR-26a promotes the axonal growth from hippocampus neurons and the axon regeneration of the optic nerve. Furthermore, we showed that miR-26a modulates axon growth by repressing the PTEN protein. Collectively, we demonstrated that miR-26a‒PTEN pathway regulates axon regeneration in mammalian CNS.

## Materials and Methods

### Animals

All 4-week-old female mice used in this study were handled according to the protocols approved by the Animal Committee of the Institute of Zoology, Chinese Academy of Sciences (Beijing, China). The miR-26a-OE^fl/fl^ mice were provided by Dr. Xiang-Hui Fu (Sichuan University, Chengdu, China). To generate miR-26a transgenic mice, a genomic deoxyribonucleic acid (DNA) fragment encoding the miR-26a-1 locus, preceded by the synthetic CAG promoter and a loxP-flanked Neo-STOP cassette, was inserted into the Rosa26 locus. Mice were generated by injecting targeted ES cells into blastocysts and maintaining in mixed C57BL/6 and 129 backgrounds. The mice carrying the targeted allele were bred with hypoxanthine–guanine phosphoribosyl transferase-Cre mice, which caused the deletion of the Neo-STOP cassette early during embryogenesis, including the germline [15,16]. The miR-26a-OE^fl/fl^ mice were crossed with CaMKIIα-Cre mice (Jackson lab, West Grove, USA) to generate miR-26a overexpression mice. The mice were housed at constant temperature (23°C) with a 12-h light/dark cycle. Genotyping was performed on tail genomic DNA.

### Reagents and antibodies

The following antibodies were used in this study: mouse anti-Tuj1 (E10045BF; Covance, Princeton, USA), rabbit anti-PTEN (mAb#9559; Cell Signaling Technology, Beverly, USA), chicken anti-GFP (A10262; Invitrogen, Carlsbad, USA), mouse anti-β-actin (A5316; Sigma-Aldrich, St Louis, USA), and mouse anti-GAPDH (glyceraldehyde-3-phosphate dehydrogenase) (AF1186, 1:2000; Beyotime, Shanghai, China) antibodies. The secondary antibodies include Alexa Fluor 488 or 568 and were purchased from Life Technology Molecular Probes, Inc. (Carlsbad, USA). The pAAV-CMV-Cre and pAAV-CMV-GFP virus vectors (titer 1.0×10^12^) were purchased from the HANBIO Company (Shanghai, China). The mouse PTEN small interfering RNA (siRNA) mixture (siPTEN ON-TARGET plus) was purchased from Thermo Scientific Dharmacon (Chicago, USA). The sequences are 5′-UGAUGAUGUAGUAAGGUUU-3′, 5′-GCGCUAUGUAUAUUAUUAU-3′, 5′-GUAGUAGGCUCAAAUAUAC-3′, and 5′-GUUACAAGUUACAUGUUUA-3′. The microRNAs and miR-26a inhibitors were purchased from GenePharma (Suzhou, China). The miR-26a sequence is 5′-UUCAAGUAAUCCAGGAUAGGCU-3′. The sequence of miR-26a inhibitor is complementary to miR-26a, 5′-AGCCUAUCCUGGAUUACUUGAA-3′.

### Primary neuron culture and *in vitro* transfection

Axon growth experiments were performed following the previously published protocols [17–19]. Briefly, the hippocampal tissues were dissected from the embryonic day 18 mouse brain, washed with ice-cold 1× PBS and digested with TrypLE Express (1×; Thermo Fisher Scientific, Waltham, USA) at 37°C for 5 min. After the tissues were washed three times with Minimum Essential Medium (MEM) plus 10% fetal bovine serum (FBS), the cells were dissociated by trituration using a 1-ml pipette tip in the culture medium. The coverslips were coated with poly-D-lysine (100 μg/ml) and laminin (10 μg/ml).

Plasmid and RNA oligos were transfected into neurons using the Nucleofector™ from Lonza (Basel, Switzerland) as previously described [20,21]. Briefly, neurons suspended in culture medium were centrifuged to remove the supernatant, and then, 100 μl Amaxa electroporation buffer with plasmid (10 μg plasmid for one transfection) or RNA oligos (6 μg RNA for one transfection) was added. Suspended cells were then transferred to a 2.0-mm cuvette and electroporation was carried out. After electroporation, the cells were immediately mixed with the pre-warmed culture medium and plated on the cell culture coverslips. After neurons were fully attached to the coverslips (about 4 h later), the cell culture medium was changed to remove the remnant toxic transfection buffer.

### Immunocytochemistry and image analysis

Cultured neurons were fixed with 4% paraformaldehyde (PFA) at room temperature for 20 min. Fixed cells were washed with PBS and blocked in blocking solution (2% bovine serum albumin, 0.1% Triton X-100, and 0.1% sodium azide in PBS) for 1 h. Primary and secondary antibodies were diluted in blocking buffer, and the cells were incubated with the desired primary and secondary antibody for 1 h at room temperature, respectively. After the immunostaining, neurons were observed with a fluorescence microscope (Zeiss Axiovert 200; Carl Zeiss MicroImaging, Inc., Oberkochen, Germany).

For embryonic hippocampus neurons, axonal trajectory was manually traced with the ‘measure/curve’ application of AxioVision software, and the lengths of axons were recorded. For the quantification of axon length, we restricted the analysis to neurons with neurite processes longer than one cell body diameter. The mean and standard error of the mean (SEM) of axon length were calculated from three independent experiments.

### Optic nerve crush and intravitreal injection

Under anesthesia (ketamine/xylazine: 100/10 mg/kg), the optic nerve was exposed intravitreally and crushed with fine forceps for 5 s at about 1.0 mm behind the optic disc. For intravitreal injection, 4-week-old female mice were anesthetized with ketamine/xylazine, and then, 1 µl AAV-virus (1.0×10^12^) or Alex 555-conjugated cholera toxin beta-subunit (CTB-555, 2 mg/ml) was injected into the vitreous body with a fine glass needle to avoid damage to the lens.

### Immunofluorescence of whole retina

Whole retinas were dissected out and fixed in 4% PFA overnight. Retinas were blocked in PBS containing 10% FBS, 0.3% Triton X-100, and 1% Tween-20 for 1 h and then incubated with anti-Tuj1 (1:250) antibody (diluted in blocking buffer) at room temperature for 10 h. After being washed 3 times (1 h for each) with PBS, retinas were incubated with the secondary antibody for 2 h at room temperature and washed again with PBS. Finally, the retinas were mounted onto slides with mounting medium H-1400 (Vector Labs, Burlingame, USA). Eight random field images were acquired per retina, and Tuj1-positive cells were counted using ImageJ software. The survival rate was calculated by measuring the number of Tuj1-positive cells in a given area of the retina as previously described [22].

### Analysis of optic nerve regeneration

The numbers of CTB-555-labeled regenerating axons were counted as described previously [22]. Briefly, to quantify the number of regenerating axons in each optic nerve, optic nerves were cut into 8-µm-wide longitudinal sections. The number of CTB-labeled axons was counted at four distances (250, 500, 750, or 1000 µm) from the injury site. By counting the number of axons and measuring the width of the longitudinal section, we calculated the axon numbers crossing per millimeter, and the average values of all the sections were obtained.

### Western blot analysis

Cortex and hippocampus tissues were quickly dissected from the mice brain in ice-cold saline and lysed in ice-cold RIPA (radio immunoprecipitation assay) buffer (Beyotime) with protease inhibitor (Roche, Basel, Switzerland). The protein concentration was determined using BCA protein assay kit (Biomed, Beijing, China). Protein samples were separated on 10% sodium dodecyl sulfate–polyacrylamide gel electrophoresis gels (Bio-Rad, Hercules, USA) and transferred onto PVDF (poly vinylidene fluoride) membranes (Millipore, Billerica, USA). After being blocked with 5% milk in TBS-T (Tris Buffered saline Tween, 0.05% Tween-20), the membranes were incubated overnight at 4°C with primary antibodies against target proteins. Then, the membranes were washed with TBS-T and probed with horseradish peroxidase-coupled secondary antibody at room temperature for 1 h. Target proteins were detected using enhanced chemiluminescent reagent (Pierce, Rockford, USA) and quantified using ImageJ software (NIH, Bethesda, USA). Quantification of protein was normalized using GAPDH.

### Quantification of mature microRNA

The level of miR-26a in cultured neural stem cells, neurons, and tissues was detected by quantitative real-time polymerase chain reaction (qRT-PCR). The experiment was conducted following the procedure described in previous studies [16,18]. Briefly, total RNA was isolated using TRizol reagent (Invitrogen, Carlsbad, USA), and thereafter, miR-26a and RNU6B were reverse transcribed using sequence-specific primers and Moloney murine leukemia virus reverse transcriptase (Roche Applied Science, Indianapolis, USA). To quantify the level of miR-26a by reverse transcriptase-PCR, aliquots of single-stranded complementary DNA (cDNA) were amplified with gene-specific primers, and RNA was reverse transcribed into cDNA with TransScript One-Step gDNA Removal and cDNA synthesis Kit (Roche Applied Science). The cDNA was analyzed by qRT-PCR) using a SYBR® Premix Ex Taq™ (Tli RNaseH Plus; TaKaRa, Dalian, China). The PCRs contained 20 ng of cDNA, Master Mix (Roche), and 200 nM forward and reverse primers in a final reaction volume of 20 μl. Each sample was run in triplicate. The analysis of qPCR of miR-26a was calculated using the 2^-ΔΔCt^ method with *RNU6B* as the endogenous control and calibrated to the control samples. The sequences of the miR-26a primers used were sequence-specific reverse transcription, 5′-GTCGTATCCAGTGCAGGGTCCGAGGTATTCGCACTGGATACGACAGCCTAT-3′; forward, 5′-GAGTGTTTCAAGTAATCCAGG-3′; and reverse, 5′-GCAGGGTCCGAGGTATTC-3′. The sequences of the *RNU6B* primers used were as follows: forward, 5′-CTCGCTTCGGCAGCACA-3′; reverse, 5′-AACGCTTCACGAATTTGCGT-3′. The sequence-specific reverse transcription primer of *RNU6B* was the same as its reverse PCR primer. For *CaMKIIα-Cre*, four sequences (P1, 5′-GCGGTCTGGCAGTAAAAACTATC-3′; P2, 5′-GTGAAACAGCATTGCTGTCACTT-3′; P3, 5′-CTAGGCCACAGAATTGAAAGATCT-3′; P4, 5′-GTAGGTGGAAATTCTAGCATCATCC-3′) were used.

### Statistical analysis

All experiments were performed three times independently. Statistics were performed using GraphPad prism software, and data were shown as the mean ± SEM. Two-tailed Student’s *t*-test was used to determine the statistical significance between different experimental groups, which was set at a value of *P*<0.05.

## Results

### miR-26a is highly expressed in hippocampal neurons and promotes its axon growth

**Figure 1.**
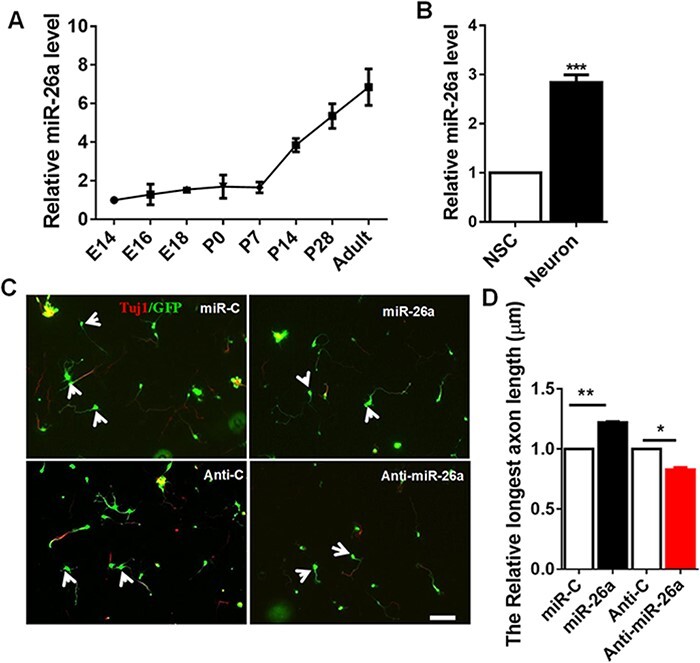
**miR-26a is upregulated during hippocampal development and controls axon growth of hippocampal neurons** (A) Relative miR-26a expression levels in mouse hippocampal tissues during development from embryo 14 days to adult (*n*=3 brain). (B) miR-26a expression levels in isolated neural stem cells and neurons. miR-26a expression was normalized to that of *U6B* small nuclear RNA gene (RNU6B) (*n*=3, ***P<0.001, Student’s *t*-test). (C) Hippocampal neurons were transfected with EGFP (control) or miR-26a mimics and were fixed at 4 days *in vitro* for axon growth analysis. Neurons were immunostained with the neuronal marker Tuj-1 (red). Scale bar: 200 μm. (D) Quantification of the axon length of transfected neurons in C (*n*=6 brain, *P<0.05, **P<0.01, Student’s *t*-test).

Our previous study revealed a novel miR-26a‒GSK3β‒Smad1 signaling pathway in the regulation of mammalian axon regeneration in the peripheral nerve system [14], which indicated that miR-26a is a vital regulator for axon growth in the peripheral nerve system [14]. To further prove the roles of miR-26a in mammalian CNS axon growth, we firstly investigated whether miR-26a is expressed in hippocampal neurons or not. Using mature microRNA-specific qRT-PCR, we found that the mRNA level of endogenous miR-26a was upregulated during developmental process, and it was highly expressed in adult hippocampal tissues as well (**Fig. 1A**). Then, we compared miR-26a expression levels between the neural stem cells and the hippocampus neurons, and the results showed that miR-26a was significantly highly expressed in hippocampus neurons than in the neural stem cells. These data indicate that miR-26a may play an important role in CNS neurons (**Fig. 1B**). To explore the function of miR-26a in CNS neurons, we transfected hippocampal neurons with the miR-26a mimics, which are double-stranded oligonucleotides designed to mimic the function of endogenous mature microRNA, or the miR-26a inhibitor, which is RNA oligonucleotides with a novel secondary structure (hairpin) designed to inhibit the biogenesis of endogenous microRNAs. Functionally, transfection of the miR-26a mimics significantly promoted axon growth, while the expression of the miR-26a inhibitor impaired axon growth (**Fig. 1C**,**D**). Taken together, our results revealed that miR-26a regulates axon growth in mammalian CNS neurons.

### Neuron-specific overexpression of miR-26a promotes axon growth in hippocampal neurons

**Figure 2.**
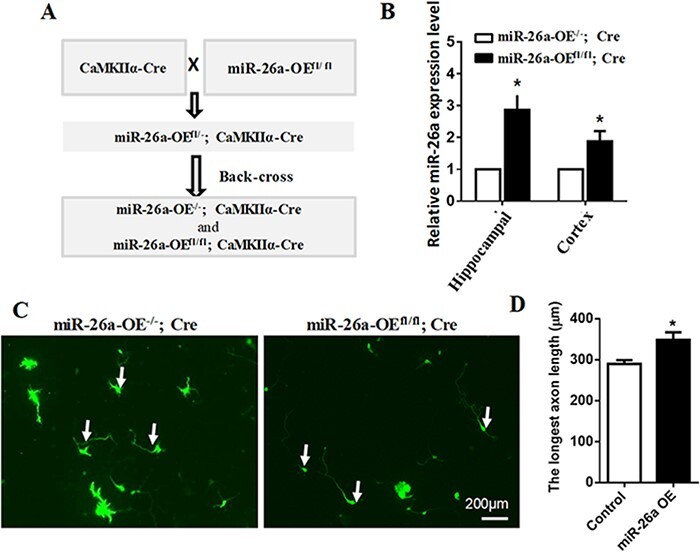
**Neuron-specific overexpression of miR-26a promotes axon growth in hippocampal neurons** (A) Schematic images of the method used to specifically overexpress miR-26a in neurons by crossing the CaMKIIα-Cre mouse with the miR-26a^fl/fl^ mice. (B) miR-26a expression levels in control (the miR-26a^fl/-^; CaMKIIα-Cre mice) and miR-26a-OE^fl/fl^; Camk2a-Cre mice. miR-26a expression was normalized to that of *U6B* small nuclear RNA gene (RNU6B). (*n*=3 mice, *P<0.05, Student’s *t*-test). (C) Images of control neurons and miR-26a overexpression neurons from miR-26a-OE^fl/fl^; CaMKIIα-Cre mice. Scale bar: 200 μm. (D) Quantification of axon length. Overexpression of miR-26a markedly promoted axon growth in cultured hippocampal neurons (*n*=3 mice, *P<0.05, Student’s *t*-test).

To further confirm the role of miR-26a in CNS axon growth, we crossed homozygous miR-26a-OE^fl/fl^ mice with transgenic CaMKIIα-Cre mice [15], to generate miR-26a-OE^fl/fl^, CaMKIIα-Cre mice. The endogenous CaMKIIα locus directs the expression of Cre recombinase specifically in the brain neurons, which leads to miR-26a overexpression in the neocortex and hippocampal neurons (**Fig. 2A**). To test the specific expression level of miR-26a in CNS neurons, the cortical and hippocampus tissues derived from the 3-week-old miR-26a-OE^-/-^; CaMKIIα-Cre and miR-26a OE^fl/fl^; CaMKIIα-Cre mice were collected. We found that the miR-26a was highly expressed in cortical and hippocampus tissues of the miR-26a OE^fl/fl^; CaMKIIα-Cre mice, compared to that in the control mice (**Fig. 2B**). Then, to investigate the intrinsic function of miR-26a in the modulation of axon growth, the E18 hippocampal neurons were cultured. Four days later, we analyzed the longest axon length of cultured neurons. The results showed that the hippocampal neuronal axon length in the miR-26a OE^fl/fl^; CaMKIIα-Cre mice was much longer than that in the control mice (**Fig. 2C**,**D**). These data further confirmed that miR-26a is a critical regulator of intrinsic axon growth ability in the CNS neurons.

### PTEN acts as a downstream target of miR-26a to modulate axon growth

**Figure 3.**
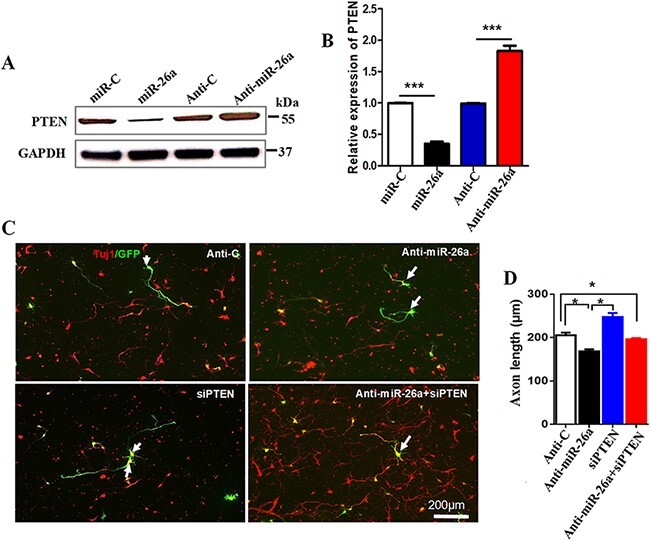
**PTEN acts as a downstream target of miR-26a to control axon growth** (A) Western blot analysis of the expression levels of PTEN after miR-26a overexpression or miR-26a inhibition. (B) Quantification of PTEN expression in A (*n*=6 mice, ***P<0.001, Student’s *t*-test). (C) Representative images of cultured hippocampal neurons. After transfection with EGFP and miR-26a mimics RNA oligos into the hippocampal neurons, neurons were immunostained with the neuronal marker Tuj-1 (red). Scale bar: 200 μm. (D) Quantification of axon growth length (*n*=3, *P<0.01, one-way analysis of variance).

The PTEN has been shown the target of miR-26a in many biological processes [23–25], and PTEN is an important intrinsic regulator of the CNS axon growth [26]. Therefore, we speculate that PTEN protein may be the downstream target of miR-26a in CNS neurons. To test this hypothesis, we first assessed the changes of PTEN protein levels after miR-26a modulation. We electroporated the miR-26a and its control miR-C and anti-miR-26a and its control anti-miR-C into the hippocampal neurons, respectively. Four days later, western blot analysis showed that the PTEN protein level was significantly reduced in hippocampal neurons with miR-26a overexpression, and it was increased after miR-26a inhibition compared to the control groups (**Fig. 3A**,**B**).

To further verify PTEN as a downstream molecule of miR-26a during axon growth, we tested that whether knockdown PTEN with siRNA can reverse the miR-26a inhibitor’s effect on axon growth. We transfected the miR-26a inhibitor, siPTEN, and EGFP (enhanced green fluorescent protein) plasmid into the hippocampal neurons. The siPTEN were four different siRNAs designed to minimize the off-target effects. Similarly, the longest axon length was analyzed 4 days later. The results showed that knockdown of endogenous PTEN alone significantly promoted axon growth (**Fig. 3C**,**D**). Importantly, we further found that miR-26a inhibitor-induced axon growth blocking effect was rescued by the PTEN deficiency (**Fig. 3C**,**D**). These results further indicated that PTEN is the downstream target of miR-26a in hippocampal neuronal axon growth.

### miR-26a improves retina ganglion cell survival and axon regeneration after optic nerve injury

**Figure 4.**
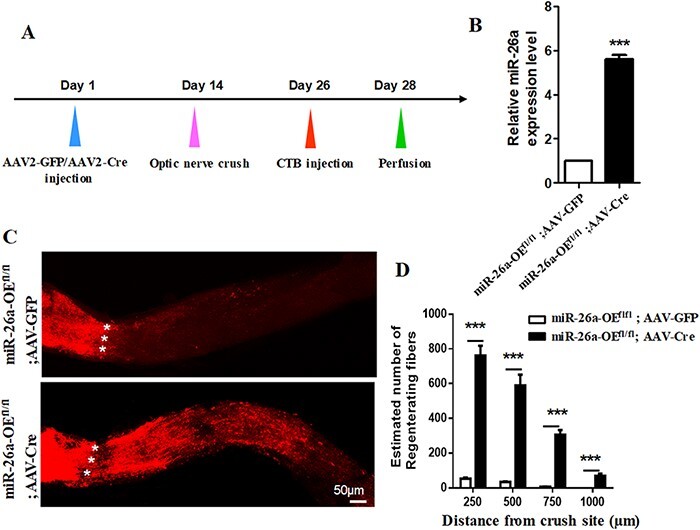
**miR-26a overexpression promotes RGC axon regeneration** (A) Timeline images of the experimental procedure. Fourteen days after AAV2-Cre (for control AAV2-GFP) virus injection, the optic nerve crush injury was performed. Another 12 days later or at day 26, the CTB-555 was injected intravitreally to label regenerating axon fibers. Two days after CTB injection, the mice were killed and axon regeneration degree was evaluated. (B) miR-26a expression levels after AAV2-Cre injection in miR-26a-OE^fl/fl^ mice compared with that in the control AAV2-GFP injection group (*n*=6, ***P<0.001, Student’s *t*-test). (C) Optic nerve sections showing anterograde-labeled CTB-555-positive axons at 14 days after optic nerve crush. Scale bar: 50 μm. (D) Quantification of regenerating axon numbers at different distances distal to the lesion sites. At least five different sections (every fourth section) from each animal were quantified (*n*=6, ***P<0.001, Student’s *t*-test).

To further identify the *in vivo* function of miR-26a in CNS axon regeneration, we performed optic nerve crush in miR-26a transgenic mice [16,27]. The vitreous body of miR-26a-OE^fl/fl^ mice was injected with AAV2-Cre virus or AAV2-GFP virus. Two weeks after virus injection, the optic nerve was crushed, and another 2 weeks later, 2 µl of CTB was injected into the vitreous body to trace the regenerated axons. We found that AAV2-Cre injection significantly increased miR-26a expression in the retina of miR-26a-OE^fl/fl^ mice when compared to AAV2-GFP injection (**Fig. 4A**,**B**), indicating that miR-26a was successfully overexpressed in retina ganglion cells (RGCs) by AAV2-Cre. Next, we observed the axon growth of the injured optic nerve. The results showed that there were no extending axon fibers found distally beyond the crush site in AAV2-GFP injection. However, AAV2-Cre-treated miR-26a-OE^fl/fl^ mice exhibited significant enhancement in the number of regenerating axons beyond the crush site (**Fig. 4C**,**D**).

**Figure 5.**
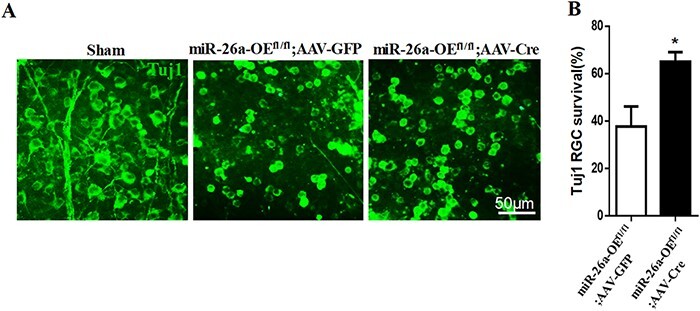
**Overexpression of miR-26a protects the RGCs from the optic nerve injury induced cell death** (A) Fluorescent photomicrographs of whole-mount retina showing the surviving Tuj-1-positive RGCs at 14 days after optic nerve injury. Scale bar: 50 µm. (B) Quantification of the number of RGCs, expressed as a percentage of the total number of Tuj-1-positive RGCs in the contralateral retina. For each retina, 15–20 fields were randomly chosen from different parts of the retina (*n*=6, *P<0.05, Student’s *t*-test).

We also analyzed whether miR-26a affects the survival of RGCs. Whole retinal tissue was immunostained using the antibody against Tuj1, a maker of neuron. Cell viability analysis revealed that more RGCs survived in AAV2-Cre-treated miR-26a-OE^fl/fl^ mice than in the AAV2-GFP injection control mice after optic nerve injury (**Fig. 5A**,**B**). Taken together, our results suggest that overexpression of miR-26a in RGCs not only promotes axon regeneration but also protects the RGC cell from the optic nerve injury-induced neuronal death.

## Discussion

Axon growth or axon regeneration is a highly coordinated process that requires multiple cellular events, including regulation of gene expression, the synthesis of essential proteins, anterograde transport of synthesized raw materials along the axon, and cytoskeletal assembly in the growth cone [28,29]. Activation of intrinsic axon regeneration ability of injured neurons is an important issue in adult animals after nerve injury. It is well known that neurons in the adult mammal CNS cannot regrow axons after injury, mainly because of their diminished intrinsic axon growth ability [18,27,29]. Therefore, understanding the intrinsic gene expression and transcription in the soma is very important to clarify how axon regrowth is regulated after injury and why axons in the mature CNS fail to regenerate [1]. Epigenetic modification, especially small non-coding RNAs, serves as the one of the major mechanisms in the regulation of gene expression, without affecting the primary genomic sequence [12]. MicroRNA is a type of short single-stranded endogenous non-coding RNA that can regulate gene expression. Many published data have shown that a bunch of microRNAs are highly expressed in the brain, such as miR-137, miR-9, miR-124, miR-26a, and miR-138 [30], and microRNAs play important roles in the regulation of neuronal function. In recent years, research on microRNAs in the mammalian CNS has focused on neurogenesis and synaptic function of mature synapses; however, few studies have explored the roles of microRNAs in post-injury axon regeneration. A recent study showed that miR-9 regulates the axon extension and branching of mouse cortical neurons by regulating the cytoskeletal protein MAP1b [31]. Our previous data also demonstrated that microRNA-138 and microRNA-26a are pivotal regulators of gene expression networks in mammalian PNS axon growth [14,18]. It was reported that miR-26a supports the axon regrowth through suppressing GSK3β activity and the subsequent expression of transcription factor Smad1 in the peripheral nervous system [14]. In this study, we found that miR-26a is also an endogenous regulator of mammalian CNS axon regeneration *in vitro* and *in vivo*. We showed that miR-26a regulates axon growth from developing either hippocampus neurons or adult RGC neurons. Our results further demonstrated that the effect of miR-26a on axon regeneration is mediated by the suppression of PTEN, suggesting that miR-26a may regulate axon growth through controlling protein translation in the neuronal soma. Our results and other published data together clearly showed that microRNAs provide a novel mechanism for enhancing axon regeneration in the nervous system.

Each microRNA usually has multiple target genes [32], and the same gene can be targeted by different multiple microRNAs, depending on the specific cellular context where the microRNA is expressed. Therefore, it has been reported that miR-26 is essential for the most complex neural activity. Gu *et al*. [33] identified that miR-26a is a regulator for long-lasting synaptic and spine plasticity and presents a catalog of candidate ‘LTP (long-term potentiation) miRNAs’ by targeting ribosomal S6 kinase 3. Using the next-generation sequencing, bioinformatics analysis, electrophysiology, and time-lapse imaging, they identified that miR-26a is essential for LTP maintenance and enlargement of dendritic spines. Moreover, one recent published paper showed that lentivirus-mediated miR-26a-modified neural stem cells improve the repair of brain injury in cerebral palsy rats through inhibiting the apoptosis of brain cells and activation of astrocytes [34]. Additionally, in non-neuronal cells, miRNAs may also be responsible for the expression of cancer-implicated genes in tumors. Huse *et al*. [35] reported that miR-26a can target PTEN in the process of glioma genesis, and miR-26a is frequently amplified at the DNA level in human glioma, most often in association with monoallelic PTEN loss. They demonstrated that miR-26a-mediated PTEN repression in a murine glioma model enhanced *de novo* tumor formation and also precluded the losses of heterozygosity and the PTEN locus. Forced expression of miR-26a in glioma cells significantly increased both growth rate and colony formation *in vitro* and tumor growth and angiogenesis *in vivo*, while reduced expression of miR-26a played opposite roles. Therefore, miR-26a has been proposed as a biomarker for glioblastoma [36–38]. All these data suggest that miR-26a may play significant roles in astrocytes, and more mechanisms underlying miR-26a in the glioma genesis need to be explored in the future.

In summary, in this study, we provide clear evidence that PTEN is a functional target of miR-26a in the hippocampal neurons to control axon growth. The overexpression of miR-26a in hippocampal neurons suppresses the endogenous PTEN level and subsequently promotes its axon growth. Additionally, overexpression of miR-26a supports axon regeneration and improves neuronal survival in RGC neurons as well. Thus, this study suggests that the miR-26a–PTEN pathway regulates CNS axon growth and provides strong evidence that microRNAs may be novel targets for promoting axon regeneration after injuries in the CNS.

## Supplementary Material

gmab044_Supp

## References

[1] Mahar M, Cavalli V. Intrinsic mechanisms of neuronal axon regeneration. *Nat Rev Neurosci* 2018, 19: 323–337. doi: 10.1038/s41583-018-0001-8 29666508 PMC5987780

[2] Zhang J, Yang D, Huang H, Sun Y, Hu Y. Coordination of necessary and permissive signals by PTEN inhibition for CNS axon regeneration. *Front Neurosci* 2018, 12: 558. doi: 10.3389/fnins.2018.00558 PMC6104488 30158848

[3] Schwab ME, Strittmatter SM. Nogo limits neural plasticity and recovery from injury. *Curr Opin Neurobiol* 2014, 27: 53–60. doi: 10.1016/j.conb.2014.02.011 24632308 PMC4122629

[4] Wang Z, Reynolds A, Kirry A, Nienhaus C, Blackmore MG. Overexpression of Sox11 promotes corticospinal tract regeneration after spinal injury while interfering with functional recovery. *J Neurosci* 2015, 35: 3139–3145. doi: 10.1523/JNEUROSCI.2832-14.2015 25698749 PMC4331631

[5] Blackmore MG, Wang Z, Lerch JK, Motti D, Zhang YP, Shields CB, Lee JK, et al. Krüppel-like factor 7 engineered for transcriptional activation promotes axon regeneration in the adult corticospinal tract. *Proc Natl Acad Sci USA* 2012, 109: 7517–7522. doi: 10.1073/pnas.1120684109 22529377 PMC3358880

[6] Lang C, Bradley PM, Jacobi A, Kerschensteiner M, Bareyre FM. STAT3 promotes corticospinal remodelling and functional recovery after spinal cord injury. *EMBO Rep* 2013, 14: 931–937. doi: 10.1038/embor.2013.117 23928811 PMC3807223

[7] Jin D, Liu Y, Sun F, Wang X, Liu X, He Z. Restoration of skilled locomotion by sprouting corticospinal axons induced by co-deletion of PTEN and SOCS3. *Nat Commun* 2015, 6: 8074. doi: 10.1038/ncomms9074 PMC4662086 26598325

[8] Liu K, Lu Y, Lee JK, Samara R, Willenberg R, Sears-Kraxberger I, Tedeschi A, et al. PTEN deletion enhances the regenerative ability of adult corticospinal neurons. *Nat Neurosci* 2010, 13: 1075–1081. doi: 10.1038/nn.2603 20694004 PMC2928871

[9] Du K, Zheng S, Zhang Q, Li S, Gao X, Wang J, Jiang L, et al. PTEN deletion promotes regrowth of corticospinal tract axons 1 year after spinal cord injury. *J Neurosci* 2015, 35: 9754–9763. doi: 10.1523/JNEUROSCI.3637-14.2015 26134657 PMC6605149

[10] Moore DL, Blackmore MG, Hu Y, Kaestner KH, Bixby JL, Lemmon VP, Goldberg JL. KLF family members regulate intrinsic axon regeneration ability. *Science* 2009, 326: 298–301. doi: 10.1126/science.1175737 19815778 PMC2882032

[11] Liu C, Teng ZQ, Santistevan NJ, Szulwach KE, Guo W, Jin P, Zhao X. Epigenetic regulation of miR-184 by MBD1 governs neural stem cell proliferation and differentiation. *Cell Stem Cell* 2010, 6: 433–444. doi: 10.1016/j.stem.2010.02.017 20452318 PMC2867837

[12] Zhang SF, Gao J, Liu CM. The role of non-coding RNAs in neurodevelopmental disorders. *Front Genet* 2019, 10: 1033. doi: 10.3389/fgene.2019.01033 PMC6882276 31824553

[13] Siegel G, Saba R, Schratt G. microRNAs in neurons: manifold regulatory roles at the synapse. *Curr Opin Genet Dev* 2011, 21: 491–497. doi: 10.1016/j.gde.2011.04.008 21561760

[14] Jiang JJ, Liu CM, Zhang BY, Wang XW, Zhang M, Saijilafu, Zhang SR, et al. MicroRNA-26a supports mammalian axon regeneration *in vivo* by suppressing GSK3beta expression. *Cell Death Dis* 2015, 6: e1865. doi: 10.1038/cddis.2015.239 PMC4558520 26313916

[15] Fu X, Jin L, Wang X, Luo A, Hu J, Zheng X, Tsark WM, et al. MicroRNA-26a targets ten eleven translocation enzymes and is regulated during pancreatic cell differentiation. *Proc Natl Acad Sci USA* 2013, 110: 17892–17897. doi: 10.1073/pnas.1317397110 24114270 PMC3816405

[16] Fu X, Dong B, Tian Y, Lefebvre P, Meng Z, Wang X, Pattou F, et al. MicroRNA-26a regulates insulin sensitivity and metabolism of glucose and lipids. *J Clin Invest* 2015, 125: 2497–2509. doi: 10.1172/JCI75438 25961460 PMC4497741

[17] Ma JJ, Xu RJ, Qi SB, Wang F, Ma YX, Zhang HC, Xu JH, et al. Regulation of adult mammalian intrinsic axonal regeneration by NF-kappaB/STAT3 signaling cascade. *J Cell Physiol* 2019, 234: 22517–22528. doi: 10.1002/jcp.28815 31102288

[18] Liu CM, Wang RY, Saijilafu, Jiao ZX, Zhang BY, Zhou FQ. MicroRNA-138 and SIRT1 form a mutual negative feedback loop to regulate mammalian axon regeneration. *Genes Dev* 2013, 27: 1473–1483. doi: 10.1101/gad.209619.112 23796896 PMC3713428

[19] Duan RS, Liu PP, Xi F, Wang WH, Tang GB, Wang RY, Saijilafu, et al. Wnt3 and Gata4 regulate axon regeneration in adult mouse DRG neurons. *Biochem Biophys Res Commun* 2018, 499: 246–252. doi: 10.1016/j.bbrc.2018.03.138 29567480

[20] Hur EM, Saijilafu, Lee BD, Kim SJ, Xu WL, Zhou FQ. GSK3 controls axon growth via CLASP-mediated regulation of growth cone microtubules. *Genes Dev* 2011, 25: 1968–1981. doi: 10.1101/gad.17015911 21937714 PMC3185968

[21] Hur EM, Yang IH, Kim DH, Byun J, Saijilafu, Xu WL, Nicovich PR, et al. Engineering neuronal growth cones to promote axon regeneration over inhibitory molecules. *Proc Natl Acad Sci USA* 2011, 108: 5057–5062. doi: 10.1073/pnas.1011258108 21383151 PMC3064397

[22] Ma JJ, Ju X, Xu RJ, Wang WH, Luo ZP, Liu CM, Yang L, et al. Telomerase reverse transcriptase and p53 regulate mammalian peripheral nervous system and CNS axon regeneration downstream of c-Myc. *J Neurosci* 2019, 39: 9107–9118. doi: 10.1523/JNEUROSCI.0419-19.2019 31597725 PMC6855683

[23] Sun H . miR-26a promotes neurite outgrowth by repressing PTEN expression. *Mol Med Rep* 2013, 8: 676–680. doi: 10.3892/mmr.2013.1534 23783805

[24] Zeitels LR, Acharya A, Shi G, Chivukula D, Chivukula RR, Anandam JL, Abdelnaby AA, et al. Tumor suppression by miR-26 overrides potential oncogenic activity in intestinal tumorigenesis. *Genes Dev* 2014, 28: 2585–2590. doi: 10.1101/gad.250951.114 25395662 PMC4248289

[25] Trohatou O, Zagoura D, Orfanos NK, Pappa KI, Marinos E, Anagnou NP, Roubelakis MG. miR-26a mediates adipogenesis of amniotic fluid mesenchymal stem/stromal cells via PTEN, Cyclin E1, and CDK6. *Stem Cells Dev* 2017, 26: 482–494. doi: 10.1089/scd.2016.0203 28068868

[26] Park KK, Liu K, Hu Y, Smith PD, Wang C, Cai B, Xu B, et al. Promoting axon regeneration in the adult CNS by modulation of the PTEN/mTOR pathway. *Science* 2008, 322: 963–966. doi: 10.1126/science.1161566 18988856 PMC2652400

[27] Wang XW, Li Q, Liu CM, Hall PA, Jiang JJ, Katchis CD, Kang S, et al. Lin28 signaling supports mammalian PNS and CNS axon regeneration. *Cell Rep* 2018, 24: 2540–2552.e6. doi: 10.1016/j.celrep.2018.07.105 30184489 PMC6173831

[28] Liu CM, Hur EM, Zhou FQ. Coordinating gene expression and axon assembly to control axon growth: potential role of GSK3 signaling. *Front Mol Neurosci* 2012, 5: 3. doi: 10.3389/fnmol.2012.00003 PMC3272657 22347166

[29] Liu CF, Liu JL, Liu CQ, Zhou Q, Zhou YD, Zhang BY, Saijilafu. The intrinsic axon regenerative properties of mature neurons after injury. *Acta Biochim Biophys Sin* 2021, 53: 1–9. 33258872 10.1093/abbs/gmaa148

[30] Obernosterer G, Leuschner PJ, Alenius M, Martinez J. Post-transcriptional regulation of microRNA expression. *RNA* 2006, 12: 1161–1167. doi: 10.1261/rna.2322506 16738409 PMC1484437

[31] Dajas-Bailador F, Bonev B, Garcez P, Stanley P, Guillemot F, Papalopulu N. microRNA-9 regulates axon extension and branching by targeting Map1b in mouse cortical neurons. *Nat Neurosci* 2012, 15: 697–699. doi: 10.1038/nn.3082 22484572

[32] Lewis BP, Burge CB, Bartel DP. Conserved seed pairing, often flanked by adenosines, indicates that thousands of human genes are microRNA targets. *Cell* 2005, 120: 15–20. doi: 10.1016/j.cell.2004.12.035 15652477

[33] Gu QH, Yu D, Hu Z, Liu X, Yang Y, Luo Y, Zhu J, et al. miR-26a and miR-384-5p are required for LTP maintenance and spine enlargement. *Nat Commun* 2015, 6: 6789. doi: 10.1038/ncomms7789 PMC4403380 25858512

[34] Guo Q, Zhang J, Zheng Z, Li X, Wang F, Liu S. Lentivirus-mediated microRNA-26a-modified neural stem cells improve brain injury in rats with cerebral palsy. *J Cell Physiol* 2020, 235: 1274–1286. doi: 10.1002/jcp.29043 31264214

[35] Huse JT, Brennan C, Hambardzumyan D, Wee B, Pena J, Rouhanifard SH, Sohn-Lee C, et al. The PTEN-regulating microRNA miR-26a is amplified in high-grade glioma and facilitates gliomagenesis *in vivo*. *Genes Dev* 2009, 23: 1327–1337. doi: 10.1101/gad.1777409 19487573 PMC2701585

[36] ParvizHamidi M, Haddad G, Ostadrahimi S, Ostadrahimi N, Sadeghi S, Fayaz S, Fard-Esfahani P. Circulating miR-26a and miR-21 as biomarkers for glioblastoma multiform. *Biotechnol Appl Bioc* 2019, 66: 261–265. doi: 10.1002/bab.1707 30408234

[37] Kwon BK, Bloom O, Wanner I-B, Curt A, Schwab JM, Fawcett J, Wang KK. Neurochemical biomarkers in spinal cord injury. *Spinal Cord* 2019, 57: 819–831. doi: 10.1038/s41393-019-0319-8 31273298

[38] Guo P, Nie Q, Lan J, Ge J, Qiu Y, Mao Q. C-Myc negatively controls the tumor suppressor PTEN by upregulating miR-26a in glioblastoma multiforme cells. *Biochem Biophys Res Commun* 2013, 441: 186–190. doi: 10.1016/j.bbrc.2013.10.034 24140063

