# Supplementary figures and images for: miR-26a promotes axon regeneration in the mammalian central nervous system by suppressing PTEN expression
Source: Acta Biochim Biophys Sin (Shanghai). 2021 Apr 23;53(6):758–65. doi: 10.1093/abbs/gmab044 (PMC8155547; doi:10.1093/abbs/gmab044)

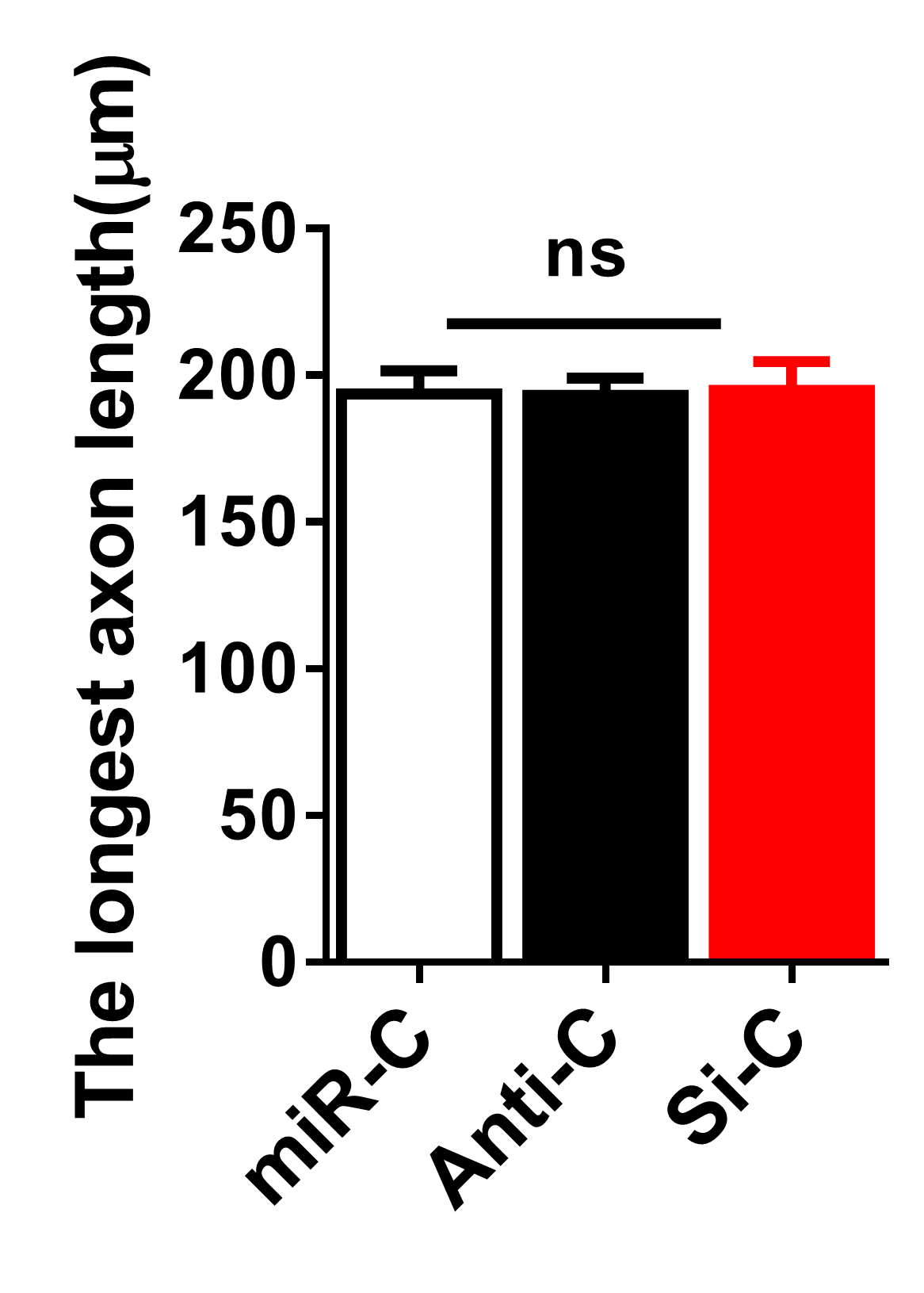

Supplement: gmab044_Supp [file gmab044_supp.zip › Supplemental Fig. S1.tif]

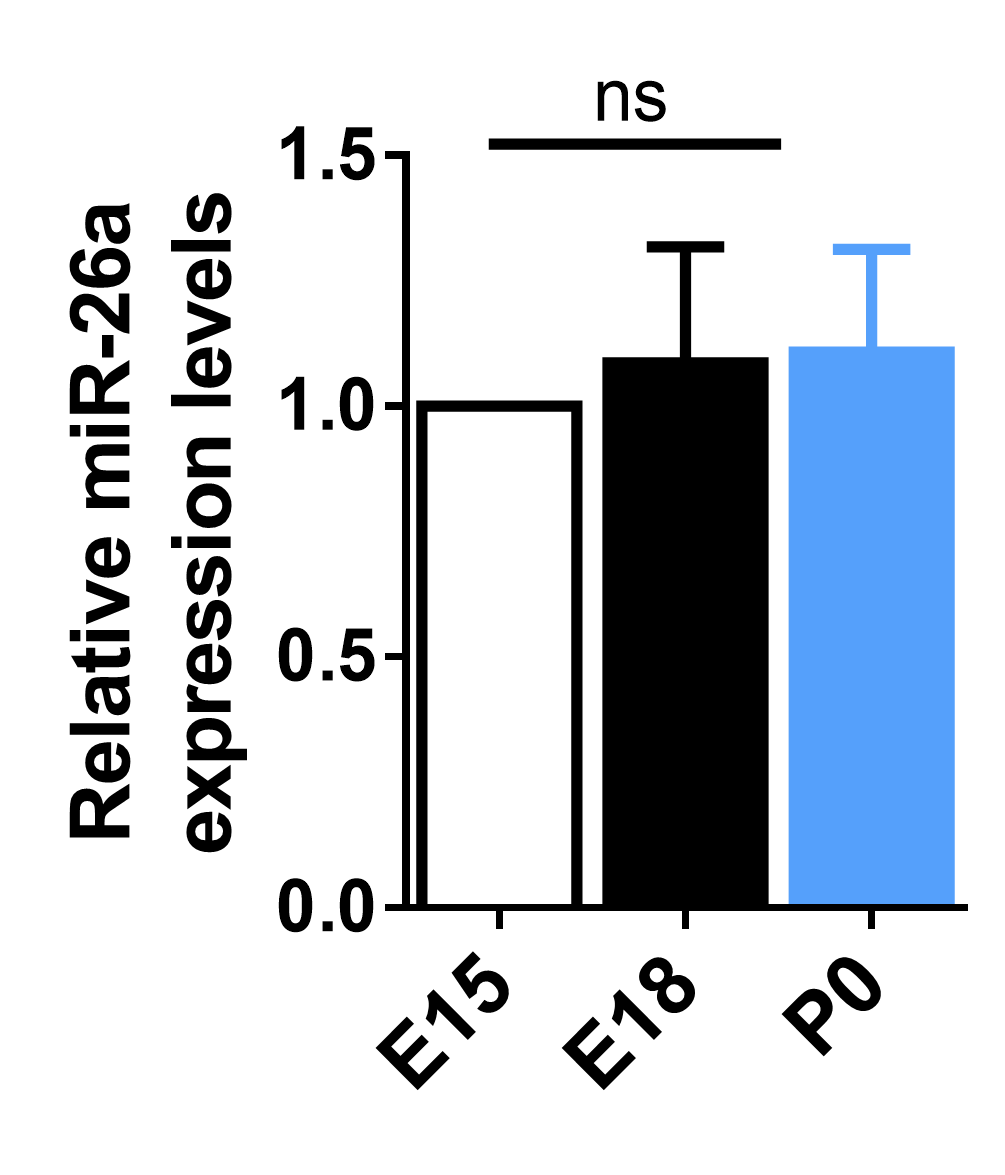

Supplement: gmab044_Supp [file gmab044_supp.zip › Supplemental Fig. S2.tif]

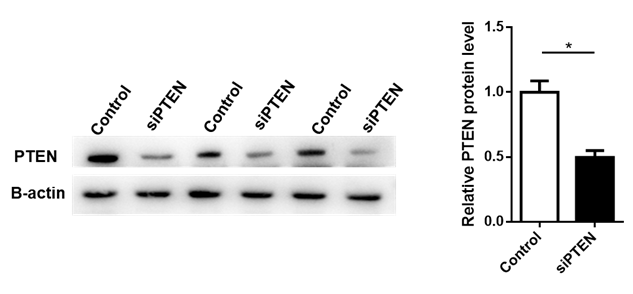

Supplement: gmab044_Supp [file gmab044_supp.zip › Supplemental Fig. S3.tif]
